# Advancing person-centered care: Protocol for quality measurement and management (QM2) in the New York State system for opioid use disorder treatment

**DOI:** 10.1371/journal.pone.0330882

**Published:** 2025-09-29

**Authors:** Sugy Choi, Sueun Hong, Adetayo Fawole, Andrew Heck, Pat Lincourt, Ashly E. Jordan, Shazia Hussain, Megan A. O’Grady, Yuhua Bao, Charles M. Cleland, Samrachana Adhikari, Magdalena Cerda, Noa Krawczyk, Kelly Kyanko, Jennifer McNeely, Chinazo Cunningham, Tod Mijanovich, Renata Howland, Olivia Thornburg, Morica Hutchinson, Edward Liebmann, Charles J. Neighbors

**Affiliations:** 1 Department of Population Health, New York University Grossman School of Medicine, New York, New York, United States of America; 2 New York State Office of Addiction Services and Supports (OASAS), Albany, New York, United States of America; 3 Department of Public Health Sciences, UConn Health, Farmington, Connecticut, United States of America; 4 Department of Population Health Sciences, Weill Cornell Medical College, New York, New York, United States of America; 5 New York University Steinhardt School of Culture, Education, and Human Development, New York, New York, United States of America; 6 New York State Psychiatric Institute, New York, New York, United States of America; PLOS: Public Library of Science, UNITED STATES OF AMERICA

## Abstract

**Introduction:**

The United States is facing an opioid use disorder (OUD) epidemic, marked by unprecedented overdose death rates. In New York State, synthetic opioids significantly contribute to the increasing overdose deaths, disproportionately impacting Black and Latinx communities. There is an urgent need to address issues related to equitable access to and the quality of care provided by substance use disorder (SUD) treatment programs. In light of this, the Quality Measurement and Management Research Center (QM2-RC) brought together an academic-government partnership to develop a person-centered quality measurement system and to assess its impact on a statewide treatment system that serves approximately 180,000 individuals per year.

**Methods and analysis:**

The QM2-RC encompasses three interconnected projects (Project 1, 2, and 3) aimed at developing a quality management strategy and evaluating its impact on system performance across New York State. This report specifically focuses on Project 3, which involves a stepped-wedge trial with 35 clinics receiving a quality management intervention that includes performance coaching. This intervention will be compared to a treatment-as-usual (TAU) condition for clinics not participating in the trial. Administrative data will be utilized to monitor outcomes over four years. The coaching intervention, guided by the Integrated Promoting Action on Research Implementation in Health Services (i-PARIHS) model, emphasizes interpreting quality measures and applying insights to enhance care. Coaches will provide support on data utilization, patient-centered care, harm reduction strategies, and the use of patient monitoring tools. The trial aims to evaluate clinic staff and leadership attitudes, experiences, and behaviors through surveys, semi-structured interviews, and external facilitator notes. Primary clinic outcomes will be assessed through adverse events, decreased clinic rates of substance use related emergency department visits and hospitalizations as well as mortality among patients within the first 12 months after admission to treatment after adjusting for individual and community level characteristics. This study is being developed over a multi-year period and will be informed by a mixed-methods approach incorporating multiple data sources, qualitative interviews, patient and clinic surveys. The study is being conducted in partnership with New York State Office of Addiction Services and Supports (OASAS) and will be informed by input from patient, providers, health insurers, family members and local governing units.

**Discussion:**

Project 3 of the QM2 study specifically targets key barriers in measuring the quality of SUD treatment, including technological limitations, unvalidated measures, workforce data literacy, and concerns about fairness in assessing clinical complexity. Through the implementation of a stepped-wedge trial involving 35 clinics, the project aims to develop new quality measures, offer performance feedback, and engage clinic leadership and staff in efforts to improve practices. The ultimate goal of Project 3 is to overcome these barriers, promote person-centered care, and improve SUD treatment practices across New York State.

## Introduction

The United States is facing an opioid overdose epidemic, with high overdose rates. In 2021, 107,622 individuals died of an overdose, which is largely associated with increasing range of synthetic substances in the illegal drug supply [1]. New York State in particular is experiencing a marked rise in synthetic opioids and overdose deaths, with nearly 5,000 attributed to opioids in 2021, accounting for approximately 68% of the increase in opioid overdose deaths between 2019 and 2021 [2]. This crisis is disproportionately affecting Black and Latinx individuals, highlighting disparities in the impact of the epidemic on communities of color [3–9]. Black and Latinx people who use drugs (PWUD) have been especially affected by the contamination of other substances with synthetic opioids [8]. The rising overdose rates [10,11] raise important questions about equitable access to treatment and the quality of care for individuals with substance use disorder (SUD) [2], particularly in high-impact areas such as New York state with a high number of opioid use and its consequences including opioid use disorder (OUD) [12–14]. OUD is associated with high morbidity and mortality including overdose death [15–21]. 

SUD treatment programs have the potential to deliver evidence-based interventions, including medication for OUD (MOUD), integrated mental health care, and recovery or supportive services, which can be lifesaving for highly stigmatized individuals such as Black and Latinx PWUD. However, previous systematic reviews have identified deficiencies in the quality of care provided by SUD treatment programs across the United States, as well as limited infrastructure and capacity to support continuous clinical improvement [22–32]. While there is variability in quality across clinics, low treatment retention [24,33–38], limited utilization of evidence-based practice [22,23] (e.g., MOUD), and disregard of patient preferences and perspectives have been widely documented [39–42].

Multiple studies have documented racial and ethnic disparities in quality of care, finding lower retention and poorer outcomes from SUD treatment for Black and Latinx patients compared to non-Latinx White patients [43–53]. Treatment programs located within communities made up predominantly of people of color tend to offer fewer and lower-quality treatment services [54]. Non-Latinx White patients clients are more likely to receive care that matches their needs [43,55]. Indeed, the most obvious reason for racial disparities in treatment outcomes may be that treatment programs are not routinely using evidence-based treatment practices [31,43,51,56–59]. For example, opioid treatment programs (OTPs) that treated a higher percentage of Black clients were more likely to dispense doses that were too low to be effective [60].

Quality measurement and reporting offers a means of holding the treatment system accountable to service users, regulators, and payors and of improving care. Despite continued calls for the development of SUD treatment quality measures, there is a dearth of generally accepted and validated measures to guide measurement and management strategies [22,27,61–63]. The National Quality Forum (NQF) recently convened two expert panels to provide recommendations on measurement development in response to the opioid epidemic [64,65]. The panel called for a quality measurement approach that is aligned with a chronic care approach to SUD/OUD treatment, with an expanded focus on treatment processes including person-centered care, measurement-based care, harm reduction, integrated care for comorbid conditions, and attention to care for special populations that are traditionally underserved, namely pregnant individuals and justice-involved populations.

The creation of actionable and implementable quality measurement and management (QM2) systems in the SUD treatment context have been hampered by three limitations. First, limited access to data and resources required to conduct measure validation has been a major barrier to developing quality measures, as requested by stakeholders [66–68]. As a consequence, SUD quality measures often rely on administrative and/or insurance claims data with a limited amount of data sources. While claims provide a readily available set of clinical data for each healthcare encounter, the billing process introduces delays in data availability, limiting the utility of quality measures for informing current quality improvement efforts [66]. In addition, claims data lack detailed information on the types of practices used for services like mental health counseling and fail to support quality measures based on patient outcomes or experience [69].

Second, SUD treatment programs’ capacities to interpret and use quality measures for clinical care improvement may be limited [22,24–28]. The SUD treatment workforce often lacks adequate education and professional training to effectively implement process change initiatives designed to enhance clinical outcomes [22,26,27,70]. An ideal QM2 study would promote the incorporation of quality measures into a culture of continued learning and adaptation [22,71]. However, limited research exists on how to best guide SUD treatment programs to use learning systems and data-driven quality improvement efforts [72,73] Research has shown that SUD treatment clinics require external support and guidance to adopt system improvement practices [74–78]. Many clinics face significant challenges in adopting these process improvement practices, including the commitment required by staff, scarce resources to implement burdensome processes, detailed data requirements, and issues with long-term sustainability [72,76,79].

Lastly, SUD treatment programs have concerns about the fairness of quality measures. One significant concern is the variability in clinical complexity, which may hinder the ability of programs to deliver consistent levels of care and potentially diminish an individual’s capacity to respond effectively to treatment [80]. Some complexities relate to health status, such as comorbid physical and mental health conditions [81–83]. Other factors affecting clinical client complexity involve social determinants of health at the person level (e.g., low educational attainment, homelessness) and the community within which the patient lives [84–86]. These complexities can create greater barriers to treatment engagement or have distinct effects on outcomes [80,87,88]. Similarly, patients living in communities with large social vulnerabilities face additional challenges that can affect their ability to benefit from treatment [89,90]. Some programs providing treatment for marginalized clients from highly stressed communities may present as performing poorly yet serve a vital role in providing services [91–93]. Consequently, programs advocate for the presentation of quality measures that are adjusted to account for the specific challenges faced by their patients and communities, such as socioeconomic factors, health disparities, and access to care.

The QM2 Study is a five-year NIH-funded center grant consisting of three projects aimed at building and testing an evidence-based QM2 study for person-centered treatment – a collaborative approach to individualized treatment. The first project of QM2 aims to develop and test a new set of quality measures for the substance use treatment system in New York State. The measure constructs will be created in conjunction with stakeholders (e.g., people who use treatment services, clinicians, and payers) to select and refine quality measures that span person-centered care, evidence-based practice, and harm-reduction approaches. This set of measures directly addresses the concerns raised in prior discussions by ensuring that quality measures are tailored to the specific challenges faced by patients and communities, accounting for factors such as patient preferences, health disparities, and access to care. The second project of the QM2 involves revising a patient-reported outcome measure for substance use disorder that was previously developed by our team. This measure is designed to capture the patient’s own perceptions of their treatment experience, including their satisfaction, engagement, and perceived outcomes. It will be refined to better reflect the diverse experiences of individuals in the treatment system, helping to assess the effectiveness of person-centered care strategies. The revised measure will play a crucial role in the QM2 study by offering insights into how well the treatment system aligns with patient needs, preferences, and goals, thereby guiding future improvements in care delivery.

This protocol pertains to the third project, which involves a stepped-wedge clinical trial. It will examine the impact of the QM2 study, incorporating new and revised measures developed in the first two projects. This study is registered on ClinicalTrials.gov under the identifier NCT06408233.

We will initiate a stepped wedge randomized controlled trial involving 1) 35 clinics receiving a performance coaching intervention and 2) a treatment as usual (TAU) condition for all clinics not participating in the intervention. Additionally, qualitative interviews with stakeholders and surveys of all 550 outpatient clinics will be conducted at three different points in time (years 1, 3, and 4). We will use administrative data from 2021–2027, including records from the Office of Addiction Services and Supports (OASAS) treatment registry, Medicaid, and vital statistics, to monitor client outcomes. This concurrent mixed-methods study [94] will evaluate the impact of the OASAS QM2 strategy on patient outcomes and statewide stakeholders, focusing on retention in care and adverse events (emergency department visits, hospitalizations, and mortality). Clinics will be randomized to one of five sequences, varying the timing of the intervention between years 2 and 4.

## Materials and methods

### Project 3 aims: Conduct a stepped-wedge trial that includes two conditions: 1) 35 clinics receiving a performance coaching intervention and 2) a treatment as usual (TAU) condition for all clinics not participating in the intervention to examine the effect of the OTP intervention on THD, retention in care, and adverse events

**Fig 1:**
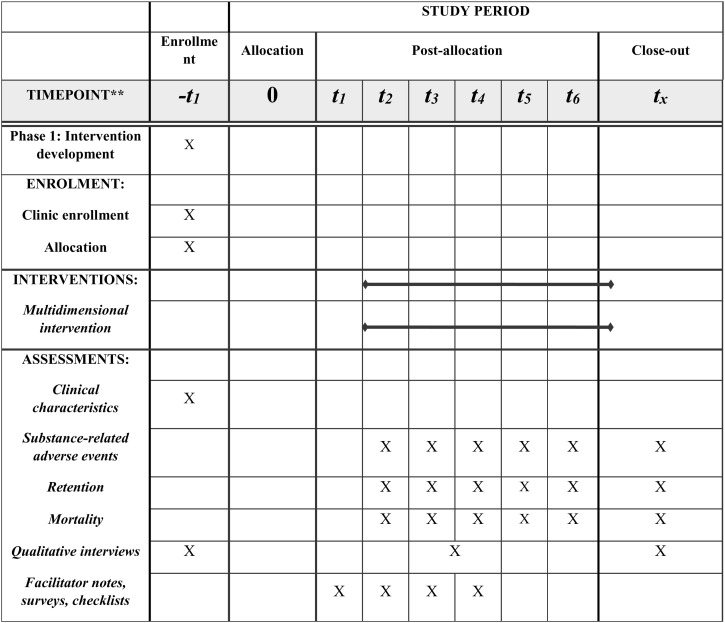
SPIRIT diagram for the QM2 Study.

The SPIRIT checklist was used as a guide for reporting this study protocol (Fig 1). To assess the impact of the OASAS QM2 strategy on care retention and adverse events using a stepped-wedge, mixed-methods design across SUD treatment clinics. Clinics will be recruited to participate in this stepped wedge trial by State partners via email invitations, announcements via State listservs, and announcements at relevant meetings to contact the Project Manager or the Principal Investigators. A total of 35 clinics will be recruited after October 2025. The aim of the intervention is to help clinical staff interpret their quality measures and implement subsequent quality improvement efforts through facilitators. The intervention will be six months long for each clinic and be informed by the i-PARIHS model [95].

i-PARIHS posits that optimal implementation occurs when practice facilitation promotes the acceptance and use of a new practice innovation by tailoring it to the recipient’s specific needs [95,96]. Facilitators are the active ingredient that help navigate individuals and teams through complex change processes by addressing a) the innovation’s degree of fit within the existing practice, b) the motivations, beliefs, goals, characteristics, and resources of the intervention recipients, and c) the inner and outer context in terms of leadership support, culture, past innovation experiences, the learning environment, organizational priorities, capacity for change, regulatory/policy drivers, incentives/mandates, and system stability/instability. Facilitators or coaches will support clinic staff examination and interpretation of the quality measures and encourage systematic adjustments to clinical and program procedures and workflows to improve care. The coaches will be trained social workers and are trained in quality improvement practices and data management as well as relevant clinical topics and will receive regular supervision by the study team. Specifically, coaches will facilitate: 1) Understanding, visualizing, and utilizing data to monitor system change and create measurable impact; 2) Providing patient-centered care, conducting patient-centered consultations and matching interventions to patient preferences, values, and goals; 3) Incorporating harm reduction strategies for SUD treatment programs; and 4) Using a patient monitoring tool for measurement-based care. The coaches will also be involved in creating training modules for the intervention.

**Fig 2:**
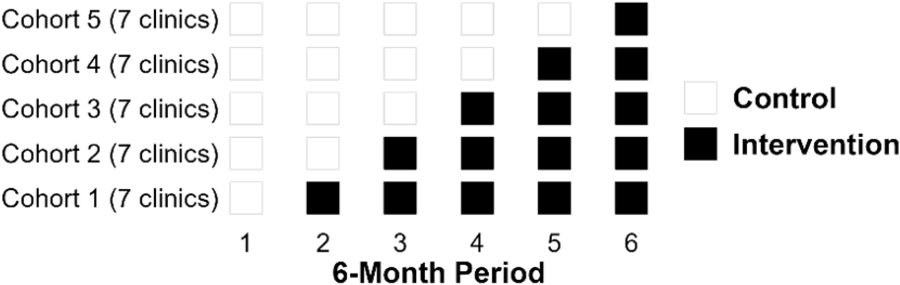
Stepped-Wedge Trial Diagram.

Before the trial begins, clinics receiving the QM2 intervention will be randomized into six cohorts of six-month long interventions over three years. Clinics are randomized to onset of intervention in one of six time periods over the three years of the intervention rollout (years 2–4 of the project) and tracked using administrative data. Administrative data will be used for all clinic clients during each of the seven six-month periods (n ≈ 350/clinic) will be drawn from administrative data. The first six-month period will be a baseline prior to any intervention. Six clinics will be assigned randomly to one of six time periods for onset of intervention. Using administrative data, we will track clients within each clinic over four years, including a pre-intervention six-month baseline period. During the same four-year period, clients in clinics not participating in the stepped-wedge trial will be tracked to better understand changes in outcomes under treatment as usual (TAU) and to compare the outcomes of those clinics with clinics receiving the quality management intervention. We anticipate a final study population of approximately 91,000 individuals per year who are receiving treatment from one of the 571 enrolled outpatient substance use treatment programs. Because this is a stepped-wedge trial, all clinics (and as a result all clients within the clinics) will eventually get the intervention, the randomization will have to do with when the clinic is randomized rather than being assigned to a control or intervention group. Fig 2 illustrates the structure of the stepped-wedge trial.

The intervention will be delivered to clinic leadership and staff. Zoom meetings with program directors will confirm enrollment and outline the timeline and expectations. We will use maximum variation purposeful sampling by recruiting clients from participating clinics in urban and rural locations as well as areas with variation in population characteristics. Clinic recruitment will be stratified by region (downstate, central, and western New York). Eligible clinics are OASAS-licensed specialty SUD outpatient programs serving at least 50 OUD clients annually. Further, we will recruit clients who are on a variety of MOUD schedules and ensure that we have representation based on gender, race, and ethnicity.

We will examine clinic staff and leadership’s perceptions of the intervention using surveys and semi-structured interviews [97–99]. The Klein implementation survey [100,101] will be administered to clinic staff at 6-months post-intervention. Semi-structured interviews (n = 72) will be conducted with at least one clinical and one member of clinic executive leadership at each clinic at the end of the six-month intervention phase. An interview guide will be created to reflect the dimensions of the i-PARIHS model [102–105]. In order to inform replication and dissemination of this intervention model, the Coaches will complete logs after each interaction with a study site [97]. Master’s-level interviewers with qualitative experience, supervised by Drs. O’Grady and Choi, will conduct all ~ 60-minute interviews. Field notes will be recorded post-interview.

### Component 1: Conduct qualitative interviews with stakeholders at years 1, 3 and 4 to examine clinic staff and leadership attitudes, experiences, and behaviors related to implementing the clinic intervention

We will recruit at least 50 participants per year in years 1, 3, and 4 of the project for 1:1 semi structured qualitative interviews until saturation is reached. Semi-structured interviews (n = 72) will be conducted with stakeholders. This includes at least one clinical and one member of outpatient executive leadership at each clinic at the end of the six-month intervention phase. An interview guide will be created to reflect the dimensions of the Health Equity Implementation Framework (HEIF), including patient-provider encounter factors from the Kilbourne framework as well as implementation factors from the i-PARIHS model. Client interviews (n = 40) from participating clinics will explore experiences related to quality of care in SUD treatment program, notably among Black/African American and Latinx clients.

### Component 2: Conduct surveys of clinics in years 1, 3, and 5 of the project to examine changes over time in the effects of the QM2 study on programs

OASAS will conduct surveys of all 550 outpatient clinics in years 1, 3, and 5 of the project. The surveys will explore change over time in provider practices (e.g., harm reduction strategies, evidence-based practices, quality improvement activities) and environmental influences related to the OASAS QM2 study (e.g., related experiences with patients/families and insurers, development of performance-based contracts). OASAS regularly surveys all clinics within its system to assess system needs. Historically, the response rate for these survey was about 60%. Surveys will be administered through the OASAS online survey data collection portal. The surveys will address the extent to which the new measures are used in quality improvement, and the burden of collecting using qualitative measures.

### Data and analytic plans

#### Project 3: Stepped-wedge trial.

*Quantitative Methods:* We will utilize Generalized Linear Mixed Models (GLMMs), which are commonly applied in the analysis of stepped-wedge trial [106,107]. The models will be structured as follows:


$$F(\mu_{ijk})=\mu +\beta_{j}+{\delta X}_{ij}+\alpha_{i}+\phi_{ik},\;\alpha_{i}\sim N\left(\text{0},\tau_{\alpha }^{\text{2}}\right),\;\gamma_{ij}\sim N(\text{0},\tau_{\phi }^{\text{2}})$$

where $\beta_{j}$ represents a fixed effect for time, $X_{ij}$ is an indicator for intervention in clinic *i* at time *j* (coded 0 for the control condition, then 1 from the start of the intervention in the clinic until the end of the study), and δ denotes the intervention effect. The models include random effects to account for clustering at both the clinic and individual patient levels, which are assumed to follow a normal distribution with mean zero and variances $\tau_{\alpha }^{\text{2}}$ and $\tau_{\phi }^{\text{2}}$, respectively. We will estimate the intervention effect while controlling for secular trends and adjusting for clustering within clinics ($\alpha_{i}$) and individual ($\phi_{ik}$), using mixed effects modeling with a binary distribution and logit link function. The models will be fitted using the *glmmTMB* package in R software [108,109]. The primary outcome will be adverse events, and the fixed-effects coefficients for the intervention effect, exponentiated, will indicate the extent to which the intervention changes the rate of adverse events. An offset term can be included to capture client differences in time at risk due to timing of treatment entry.

TAU clinics will be included in the model with an indicator variable. A post-hoc test of the intervention effect coefficient will compare TAU and performance coaching, and a TAU-by-time interaction will assess time trends within the TAU group. The GLMM analysis will be expanded by adding the clinics not receiving the performance coaching intervention during the study period. For TAU clinics, the intervention indicator will be coded 0 in all periods. This provides an additional estimate of trends ($\beta_{j}$) in outcomes in a much larger sample of clinics. Because the fixed effect for time ($\beta_{j}$) is estimated, the intervention effect (δ) can be interpreted as the degree to which performance coaching results in a lower rate of adverse events than what would be expected due to time alone in the absence of intervention.

If we observe significant treatment effects (e.g., reduced adverse events) from the performance coaching intervention, we will conduct follow-up analyses to strengthen causal inference. Drawing on methods from treatment mechanism research, we will test whether hypothesized mediators (e.g., person-centered care, retention in treatment) are associated with outcomes. We will also examine moderation by population characteristics and regional variation (e.g., gender, location), as well as provider-level differences in implementation. Lagged treatment effects will be assessed using interaction terms between treatment and time since intervention start by cohort. These exploratory analyses will inform interpretation and guide qualitative inquiry.

*Data and specification:*

We will join multiple administrative data sources from New York State for years 2021–2027 to form our analytical dataset. We will use Client Data System (CDS), Medicaid claims, and NYS vital statistics data to form the analytic dataset. The OASAS treatment registry (CDS) will provide extensive clinical data on clients at treatment admission and will include descriptions of providers through the provider data system (PDS). Medicaid will provide medical diagnoses, healthcare services, SUD treatment services, and medication utilization data. The New York State Vital Statistics will be received from the outpatient clinics in the form of all-cause mortality for clients participating in the trial. Additional OASAS data will provide operational and staffing information for each clinic. To date, 75% of clients in the OASAS registry have been linked to Medicaid claims data.

Quality measures from Project 1 will encompass patient, clinic, and organizational factors, including demographics, clinical complexity, substance use patterns, prior healthcare utilization, community access, substance use trends, resources, psychosocial interventions, clinic structure, staffing, and MOUD use and retention. Primary outcomes include adverse events, healthcare utilization, and mortality. Adverse events are defined as SUD-related hospitalizations or emergency department visits, including overdose, within 12 months of treatment entry. Healthcare utilization includes all-cause emergency department visits or hospitalizations within the same timeframe. Mortality will be assessed as all-cause mortality. Outcome variables will be risk-adjusted to account for client health status.

*Statistical Analyses of the Proposed Stepped-Wedge Trial:* Analyses will begin with examination variable distributions, appropriate transformations, and patterns for, followed by formal imputation methods if needed [110–112]. We will use Generalized Linear Mixed Models (GLMM) for the stepped-wedge design [106].

*Power Analysis for Primary Outcome Statistical power:* Power estimation for specific outcomes was conducted using PASS 2022 software and Monte Carlo simulation [113]. Sample size and baseline rates (μ) were estimated using administrative data. For 80% power, α = 0.05, ICC = 0.05, and a 5-crossover-point stepped-wedge-RCT with 7 clinics starting at each point, power calculations indicate 80% power to detect a small decrease (RR = 0.93) in adverse events with 35 clinics and 350 clients per clinic per 6-month period (n ≈ 73,500 total observations). This corresponds to a decrease from 0.23 to 0.21 events per 6 months. In racial/ethnic subgroups (10% of clients, n ≈ 35 per site), power remains 80% to detect a modest decrease (OR=0.78), from 0.23 to 0.18.

#### Component 1: Qualitative interviews.

We will use an iterative coding process, where initial stakeholder interviews develop the coding scheme, which will be refined as saturation is reached. Subsequent transcripts will be compared to ensure consistent code assignment. As new concepts emerge, the coding structure will be adapted. Grounded theory principles will guide iterative data collection and analysis, with interviews evolving based on emerging themes. Constant comparative analysis will be employed for coding and theme development, and memo writing will follow each interview. The research team will review initial codes, select core categories at data saturation, and complete advanced theoretical coding to ensure logical and comprehensive analysis [114]. After the team has reviewed the coding structure and all interviews have been reviewed in depth by two researchers, trained project staff will independently code all transcripts using the final coding scheme. Twenty percent of the transcripts will be double coded to assess inter-coder agreement. Any differences in coding will be discussed and resolved after discussion with the investigators. Data will be entered into a qualitative software program (Dedoose) [115] to facilitate organization and retrieval. We also will create an analysis audit trail to document all analytic decisions. Targeted analyses will examine the consistency of the data across groups of interviewees. When coding is complete, the team will meet to review summaries of the qualitative results and refine hypotheses about the factors affecting use of the QM2 study.

*Qualitative Analysis:* The semi-structured interviews will be transcribed and analyzed using directed content analysis to identify key facilitators, barriers, and experiences related to the performance coaching intervention [116,117].

Data will be managed using Dedoose software, with field notes taken immediately after each interview. Initial interviews will help develop the coding scheme, and subsequent transcripts will be compared to ensure consistency. As new concepts emerge, the coding structure will be adjusted. Data collection and analysis will be iterative, with interviews modified to reflect emerging themes. After reviewing the coding structure for logical consistency, two researchers will thoroughly examine all interviews before trained project staff independently code all transcripts. To assess inter-coder agreement, 20% of transcripts will be double-coded. Any coding discrepancies will be discussed and resolved with the investigators. An analysis audit trail will be created to document all analytic decisions. Targeted analyses will assess data consistency within sites and identify themes distinguishing high and low-performing clinics. Once coding is complete, the team will review qualitative summaries and refine hypotheses regarding factors and strategies that contribute to successful outcomes or present barriers. For the quantitative survey results, group means and standard deviations will be calculated.

#### Component 2: Survey data.

We will examine within interview period (i.e., cross sectional) and across time (i.e., longitudinal) variation in provider behavioral and environmental influences. Survey data will be linked to the administrative data described above so that we can model clinic variation while adjusting for clinic, client, and community characteristics. We will begin by looking at within interview period descriptive data to examine variation across clinics across survey measures. We will then apply GLMM to examine the observed variation while adjusting for clinic, client, and community characteristics. The models will take the form $F\left(Y_{ij}\right)=\;\alpha +\;\beta_{j}+\;\beta_{\text{1}}C+\;\beta_{\text{2}}P+\;\beta_{\text{3}}E+\;e_{j}$, where $Y_{ij}$ is the survey measure of interest for clinic *i* at time *j,* $\beta_{j}$ is a fixed effect for time, C is a matrix of measures for clinic characteristics, P is a matrix of measures for clinic client characteristics, and E is a matrix of measures for community factors. For examination of change in practices and environmental influences over time, we use GLMMs with random effects for clinic to account for repeated observations. The parameter of interest will be $\beta_{j}$.

### Ethics approval

Ethics approvals have been obtained from the Institutional Review Board (IRB) of NYU Langone Health, which is overseeing all participating sites: New York State OASAS, University of Connecticut (UConn Health), and Weill Cornell Medicine (WCM). The IRBs at NYU Langone Health and WCM have determined that the research conducted at WCM does not involve human subjects, thus no reliance agreement exists between these two IRBs. However, there is a reliance agreement in place with OASAS and UConn Health. All participants involved in surveys and interviews will provide written or verbal informed consent. Written consent will be collected for in-person data collection, while verbal consent will be obtained for remote or telephone-based interactions, documented by the study team in a secure tracking system, and witnessed by a second team member when possible. The use of administrative data will not require individual consent, as it will be de-identified and used in accordance with institutional and state data-sharing agreements. Retrospective data will be accessed for research purposes starting October 1, 2025, following clinic recruitment. During data collection and analysis, authors may have access to personally identifiable information. Data use agreements with OASAS ensure compliance with all relevant privacy and confidentiality standards.

### Dissemination

This study will adhere to the NIH Public Access Policy, ensuring public access to the published results of NIH-funded research. The trial has been registered with the ClinicalTrials.gov database (#NCT060823). Data generated from this study will be presented at national or international conferences and published promptly. Any published data presentations will be communicated to participants and other stakeholders involved in the trial. All final peer-reviewed manuscripts resulting from this proposal will be submitted to the digital archive PubMed Central. Additionally, the results of this project will leverage OASAS’s investment in its new QM2 strategy and policy leadership.

### Status and timeline

The stepped-wedge trial is projected to begin in July, 2025. We anticipate completing participant recruitment by October 2025, data collection by December 2026, and expect to have preliminary results available by August 2027.

## Conclusion

Quality measurement and improvement in OUD treatment are impeded by several obstacles, particularly in resource-constrained environments. These challenges include insufficient technological capacity due to limited funding, a lack of validated quality measures for specialty SUD treatment, workforce difficulties in data management resulting from inadequate training resources, and concerns from clinic leadership about the fairness of performance assessments that fail to consider the clinical complexity of their clients. To address these challenges, this study will implement the QM2 intervention, which aims to provide performance feedback to clinic leadership and staff to encourage practice improvements and to publicize quality measures for public accountability. The intervention will develop and utilize risk-adjusted quality measures that consider the clinical complexity of clients, thereby ensuring a fair evaluation of clinic performance. By tackling these barriers, the QM2 intervention seeks to promote person-centered care and contribute to the growing body of literature on effective quality improvement strategies in SUD treatment.

## Supporting information

S1 File SPIRIT Checklist. (PDF)
